# Management of Non-union Distal Femur Fractures With Augmentation Nail Plate Construct

**DOI:** 10.7759/cureus.37173

**Published:** 2023-04-05

**Authors:** Vikas Saxena, Varun Akshay, Akshay Panwar, Satendra Kumar

**Affiliations:** 1 Department of Orthopedics, Government Institute of Medical Sciences, Greater Noida, IND; 2 Department of Surgery, Government Institute of Medical Sciences, Greater Noida, IND

**Keywords:** extra-articular distal femur fractures, retrograde intramedullary femoral nail, distal femur fracture, nonunion of distal femur, nail and plate combination

## Abstract

Background and objective

A non-union distal femur fracture is a challenging fracture to treat. Common treatment modalities for non-union distal femur fractures include dual plating, intramedullary nails, ilizarov, and hybrid fixators. Despite the availability of a wide armamentarium of constructs, the clinical and functional outcome of these modalities is often complicated by significant morbidity, joint stiffness, and delayed union. The augmentation of the intramedullary nail with a locking plate results in a robust architecture, improving the likelihood of union. The use of this nail plate construct improves biomechanical stability and restores limb alignment, which enables early rehabilitation and weight bearing and lowers the likelihood of fixation failure.

Methodology

A prospective study was conducted at the Government Institute of Medical Science, Greater Noida, from January 2021 to January 2022 on 10 patients with non-union of the distal femur. All the patients were operated on with nail plate construct. The minimum follow-up period was 12 months.

Results

A total of 10 patients with a mean age of 55 years were included. Six were earlier treated with an intramedullary nail and four with extramedullary implants. All patients were managed with implant removal and fixation with nail plate construct and bone grafting. The average duration of the union was 10.3 months. The International Knee Documentation Committee (IKDC) score improved from 30.6 preoperatively to 67.3 postoperatively. Only one patient developed a superficial infection, which was managed by wound debridement and targeted antibiotic therapy.

Conclusion

In our experience, this relatively novel technique of combining nail plate constructs offers encouraging outcomes in the management of non-union of distal femur fractures, especially in elderly and osteopenic patients.

## Introduction

A non-union distal femur fracture is a challenging fracture to treat. A distinct subtype of periarticular fractures, distal femur fractures are becoming more common in elderly and osteoporotic patients [1]. They constitute 6% of all femur fractures [2], and around 50% of these are open fractures [3]. Despite the availability of a wide armamentarium of constructs, the clinical and functional outcome of these modalities is often complicated by significant morbidity, joint stiffness, and delayed union. With the breakage of intramedullary implants, the treatment of such non-union becomes even more difficult and challenging. Lateral locking plates (LLP) and retrograde intramedullary nails (rIMN) are the conventional treatment modalities for distal femur fractures, and both techniques have shown similar union rates [4,5]. Although dual plating provides a firmer fixation, a second surgical incision results in soft tissue complications, which may have a negative impact on the healing of the fracture. Fixation with either of these structures does not give patients with complex fracture patterns the biomechanical stability for early weight bearing and mobilization, especially in the presence of osteoporosis [5-9]. The use of the nail plate construct (NPC) improves biomechanical stability and restores limb alignment, which may enable early rehabilitation and weight bearing and lower the likelihood of fixation failure [10-14].

Biomechanics of nail plate construct

In contrast to the lateral locking plate, which is a load-bearing implant, the retrograde intramedullary nail transfers the weight-bearing axis toward the anatomical axis of the femur and distributes forces over the surrounding cortex and through the nail-cortical interface. Combining these provides a more robust architecture that enables early patient mobilization, improving the likelihood of union [8,9]. According to Liporace and Yoon [10], the IM implant's location initially shifts the weight-bearing axis more medially and toward the anatomical femur axis, while the LLP adds further stability to lessen movement at the fracture site. In a study using a synthetic femur with a fracture in the 33A3 shape, the nail plate combination demonstrated higher torsional as well as axial strength.

## Materials and methods

A prospective study was conducted at the Government Institute of Medical Science, Greater Noida, from January 2021 to January 2022 on 10 patients with a non-union distal femur. All the patients were operated on with nail plate construct. The minimum follow-up period was 12 months. Patients included in our study had to fulfill certain criteria, including all radiologically confirmed non-union of extraarticular fractures of the distal femur with the implant in situ above the age of 18 and being medically fit to undergo an operation. All the patients who were excluded were those who had either intra-articular fractures or pathological fractures.

All of the patients in the study were admitted from OPD of the Orthopaedics department at our hospital. Post-admission, a complete history of each patient was noted regarding the mode of injury, the severity of the trauma, the duration of the injury, and associated comorbidities. Any history regarding re-injury in the patient was also taken. A thorough examination of the limb was done, and deformity, range of movement, shortening, and ability to bear weight were assessed. A pre-injury International Knee Documentation Committee (IKDC) score was calculated.

Radiographs of the involved extremity were taken from the hip to the knee in a neutral position in the anterior and lateral positions. Radiographic parameters noted were the visibility of the fracture line and the presence of callus among the cortices. ESR and CRP were done in all the patients to rule out infection.

Implants were removed, followed by the freshening of bone margins, and then the fracture was fixed with an intramedullary nail. The fixation was further augmented by a distal femoral locking compression plate, and bone grafting was done. Post-operative functional assessment was done by evaluating decreased pain and return to function as well as radiographic evidence of fracture consolidation and the IKDC score.

Surgical technique

After antibiotic administration and adequate anesthesia, the patient is laid supine on a radiolucent operative table with a bolster under the ipsilateral knee as an aid in reduction. Under aseptic precautions, implant removal is done along with freshening of bone ends.

Retrograde Femoral Nail Insertion

Entry is made either through a stab incision through the patellar tendon or through a transverse incision through the kneecap. The entry point is placed under fluoroscopic guidance with a guide wire in AP view, centred exactly in the middle of the intercondylar notch, and in lateral view, at the extension of Blumensaat’s line. Further, the entry point is in line with the axis of the medullary canal. The femur is then reamed and a nail inserted.

Lateral Locking Plate Application

After adequate reduction of fracture fragments, a plate is applied. A longer plate is used, which allows for the proximal integration of the two constructs together. The plate is then placed, and its position is checked under fluoroscopy. After ensuring adequate alignment of the implant with respect to the joint, distal screws are applied first, followed by proximal. Distal screws are applied in such a fashion as to avoid interference with nails.

Integrating the Two Implants Together

The design of the implants, the lateral locking plate, and the retrograde intramedullary nail allow for utilizing the two with the careful application of interlocking screws. The two distal and central screw holes of the lateral locking plate that were earlier left vacant in steps prior are now interdigitated with the distal interlocking screws of the retrograde intramedullary nail. Proximally, the fixation of the construct depends on the construct that extends more proximally. In the case of a longer nail construct, the two proximal nail holes are filled in the usual manner with two bicortical screws that are aimed anterior and posterior to the nail. In the case of a longer plate, the implants can be linked using non-locking screws in the plate aimed through the holes in the nail.

Postoperative Protocol

The range of movement exercises are initiated the next day under the supervision of the physiotherapist, while weight bearing is initiated according to the fracture morphology, patient characteristics, and safety. Typically, the patients are put on partial weight bearing after stitch removal (14 days).

## Results

Ten non-union distal femur patients were included in the study conducted at Gims Hospital in Greater Noida. All patients were males within the age range of 44-70 years, with a mean age of 55 years. Six were earlier treated with intramedullary implants and four with extramedullary implants in the distal femur. All patients in our study presented with broken implants. All fractures were fixed with an intramedullary distal femoral nail, and the fixation was strengthened by a distal femoral locking plate. All patients underwent iliac crest bone grafting. All patients progressed to the union of the fractures within an average duration of 10.3 months. The IKDC score increased from an average of 30.6 to 67.3. One patient developed a superficial infection that later underwent wound debridement and antibiotics according to culture and sensitivity and healed eventually.

Case 1

**Figure 1:**
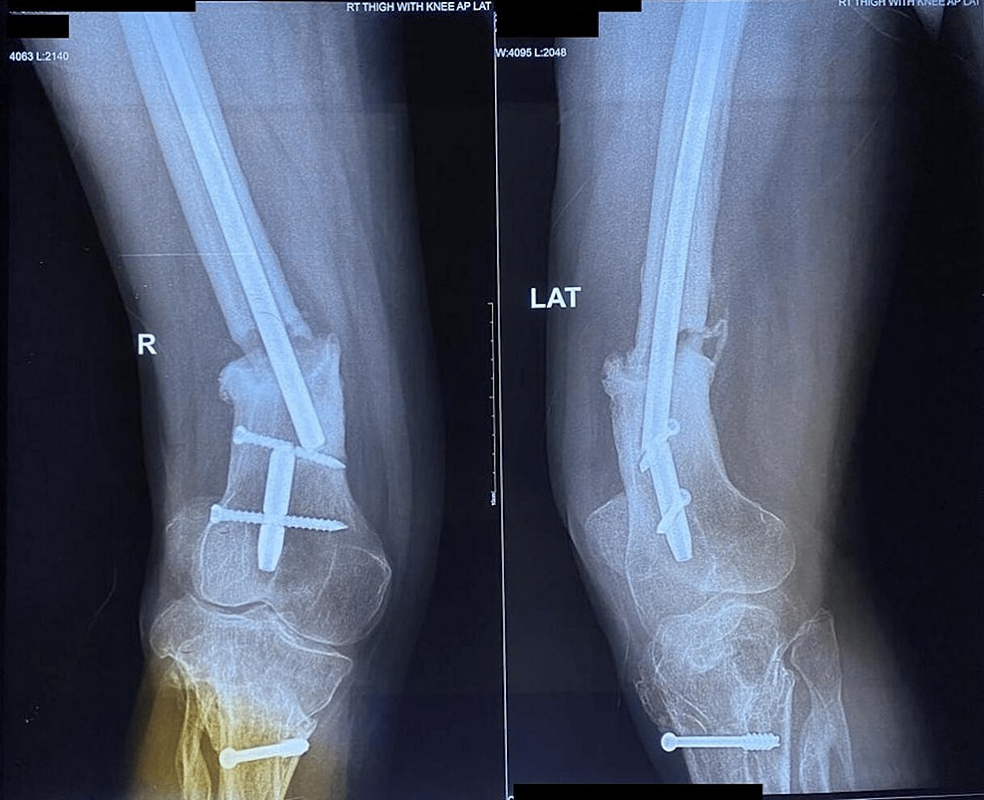
Pre-operative radiograph of patient 1 with broken intramedullary nail in situ.

**Figure 2:**
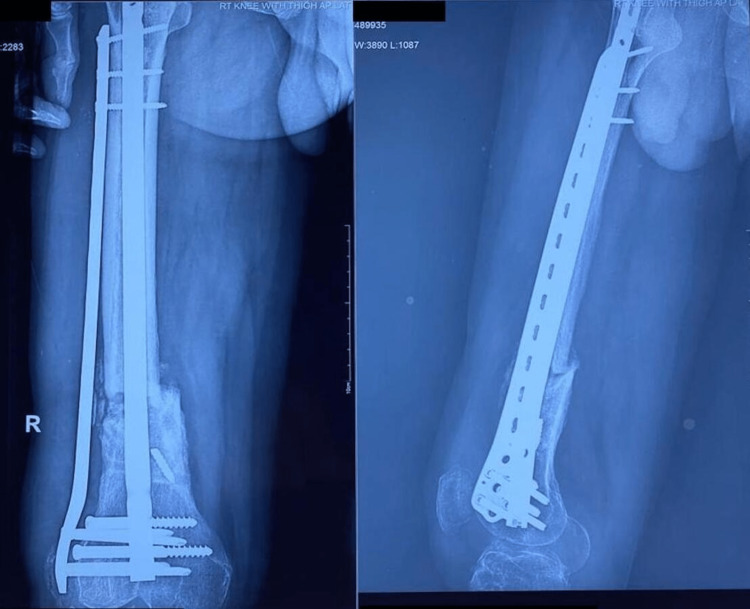
Post-operative follow-up radiograph of patient 1 at one-year follow up showing bridging callus formation.

**Figure 3:**
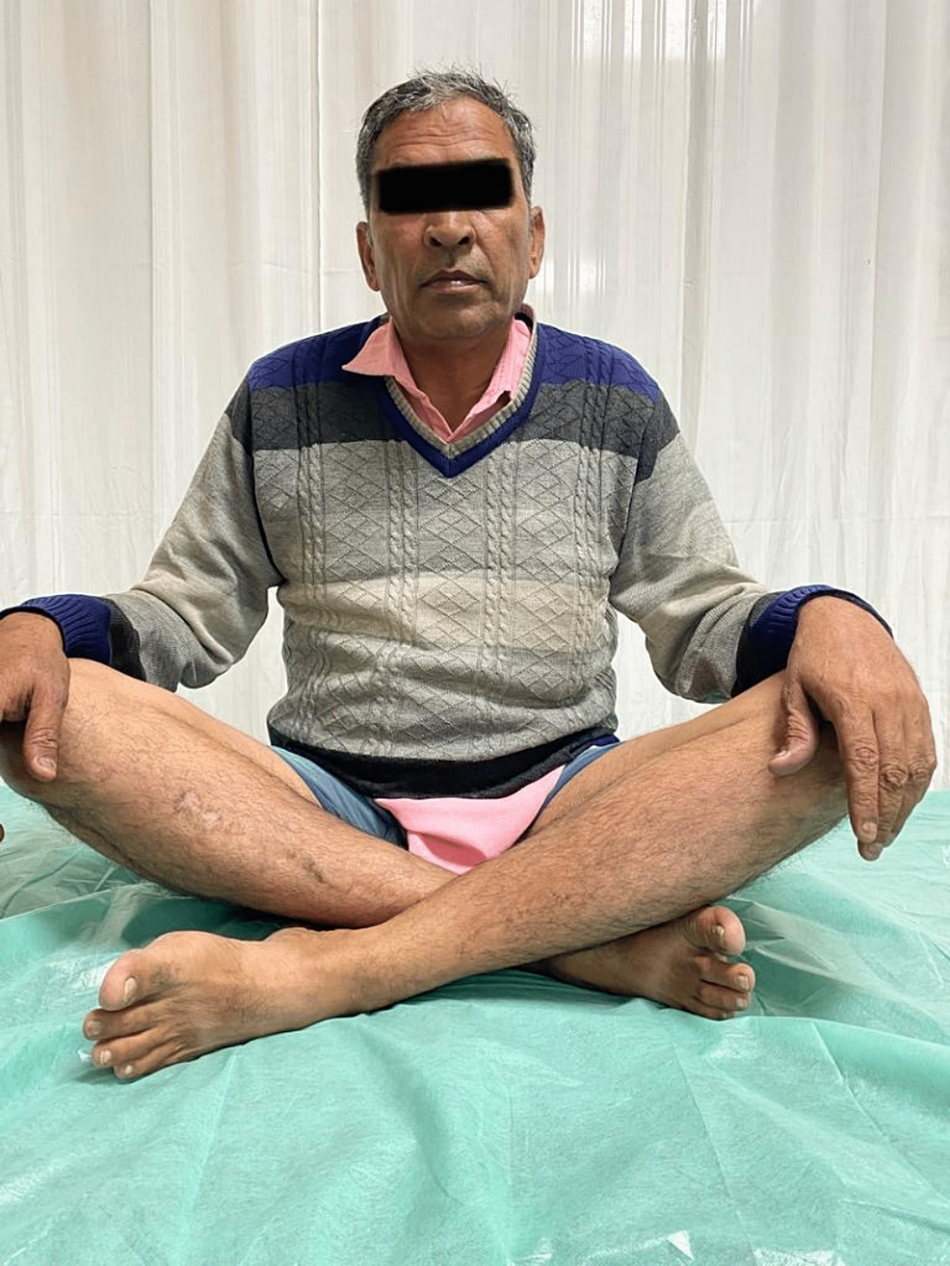
Clinical photograph of patient 1.

**Figure 4:**
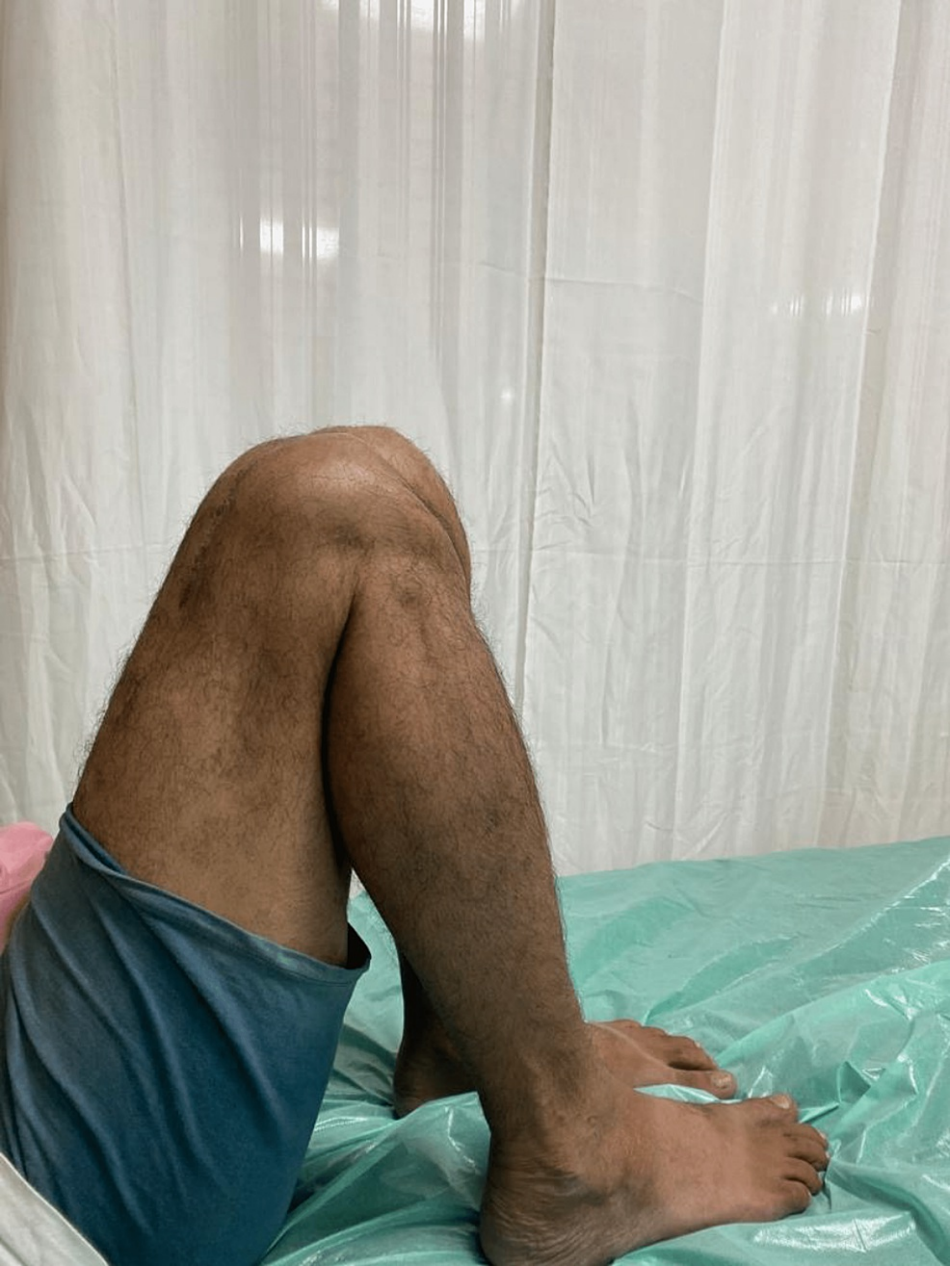
Clinal photograph of patient 1.

The patient sustained an injury to the right thigh and leg and was operated on at another centre a year ago. He presented to us with pain and deformity over the right distal thigh, for which radiographs were taken (Figure 1), which showed a non-union fracture of the shaft of the femur with a broken nail in situ. He was managed operatively with implant removal and nail plate construct along with ipsilateral iliac crest bone grafting. Radiographs at one-year post-surgery (Figure 2) show uniting fractures with bridging callus formation. The patient has an excellent range of movement one-year post-surgery, as demonstrated in Figures 3-4.

Case 2

**Figure 5:**
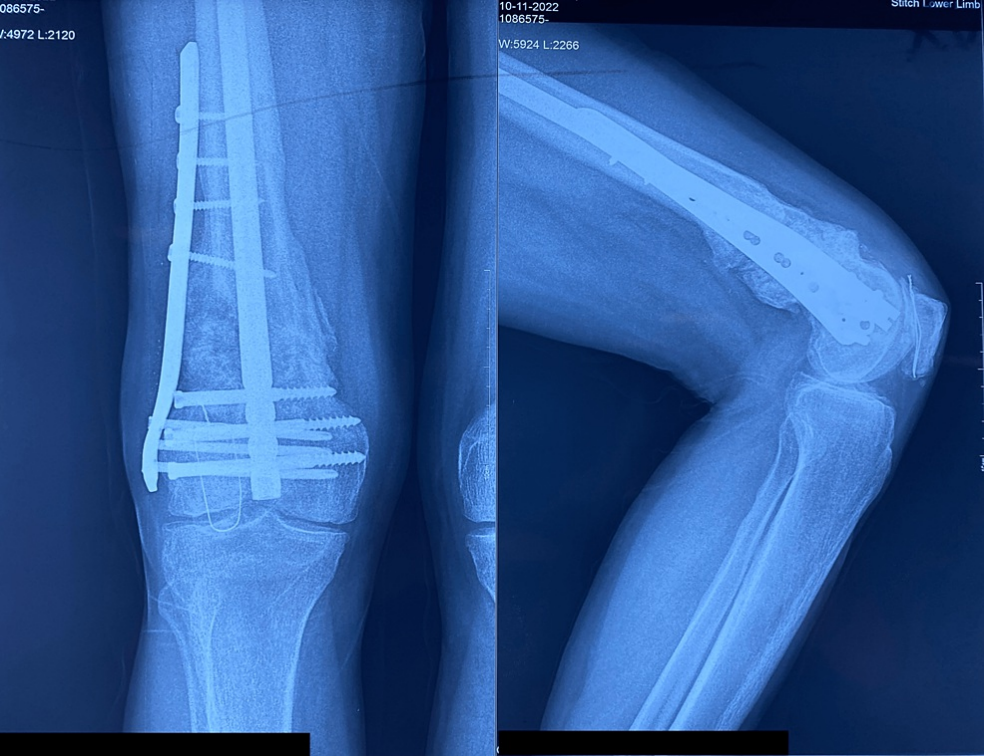
Radiograph at one-year follow up of patient 2 showing bridging callus with fracture union.

**Figure 6:**
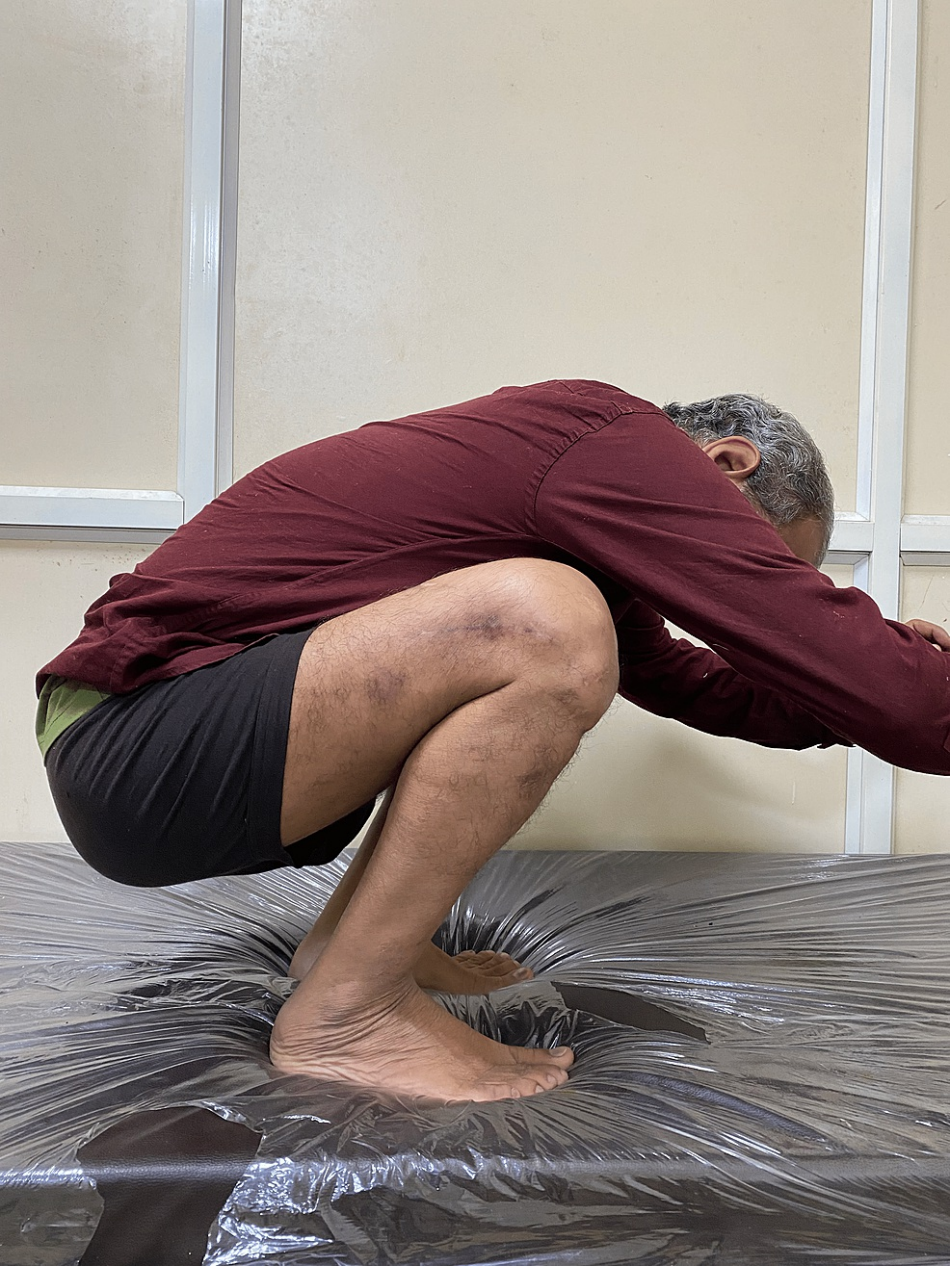
Clinical photograph of patient 2.

**Figure 7:**
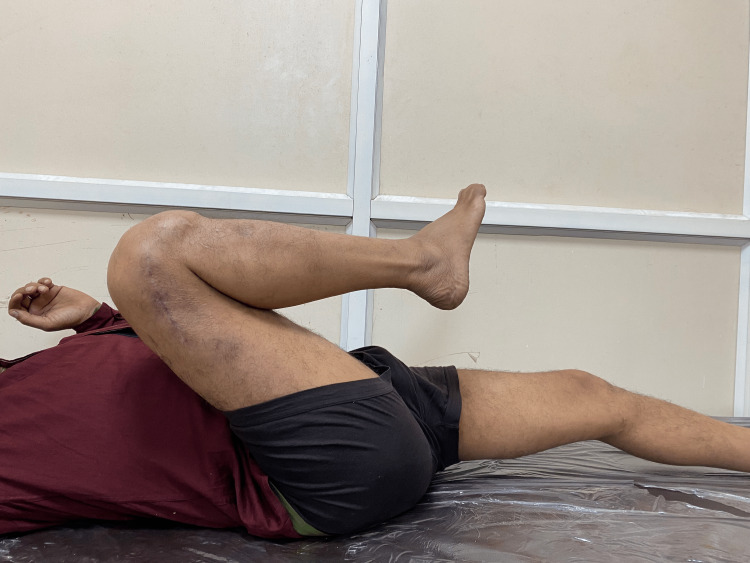
Clinical photograph of patient 2.

**Figure 8:**
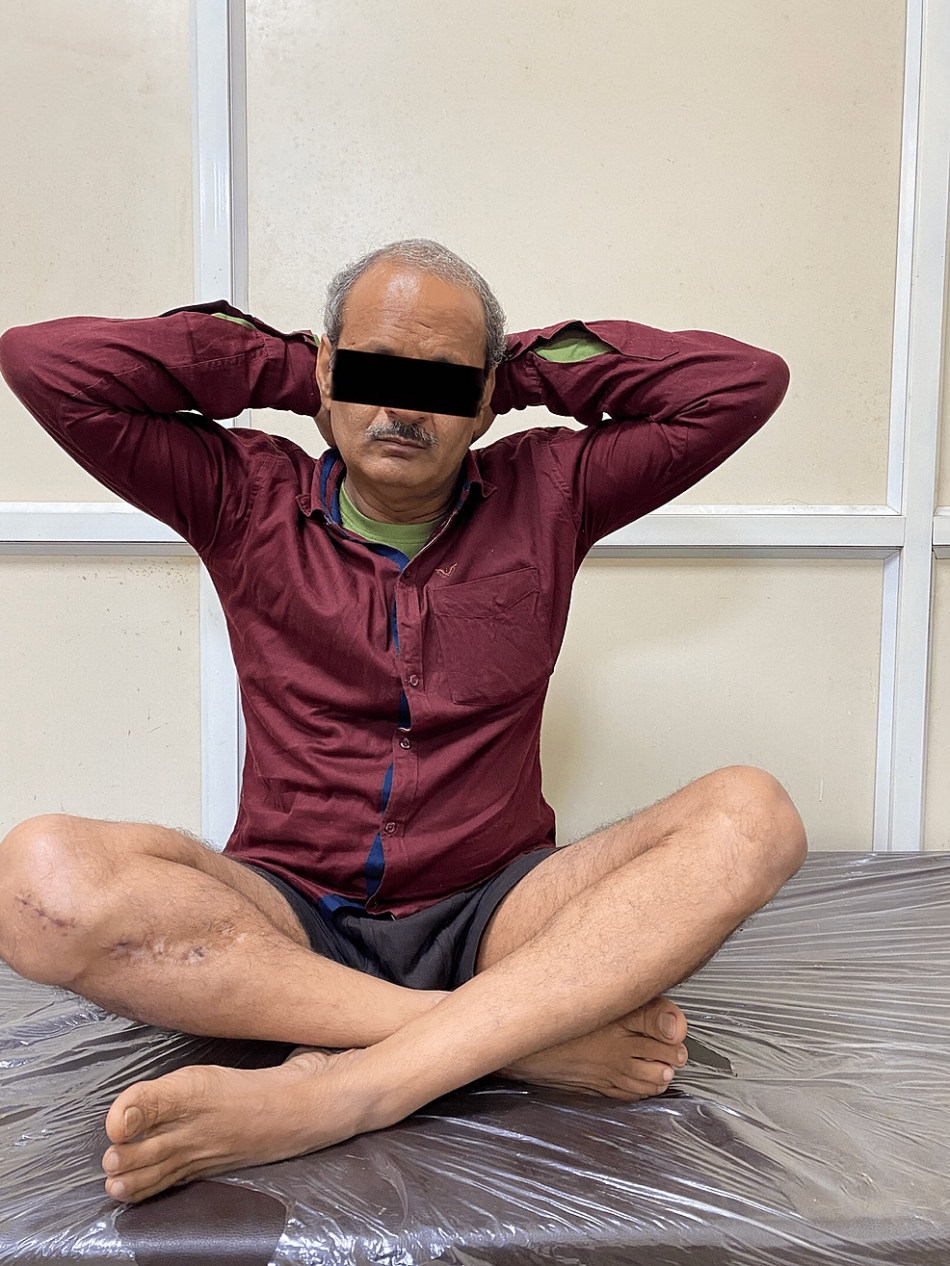
Clinical photograph of patient 2.

The patient sustained an injury to the right distal thigh and knee eight months ago, for which he was operated on at another centre. He presented to us with a history of a fall from stairs followed by pain and swelling over his right knee. A radiograph demonstrated a non-union fracture at the distal end of the femur with a broken implant in situ. He was later managed operatively with implant removal and nail plate construct along with ipsilateral iliac crest bone grafting. Radiographs at one-year post-surgery (Figure 5) demonstrate bridging callus formation. Figures 6-8 show excellent functional results at one-year follow-up.

## Discussion

The treatment of non-union distal femur fractures has evolved immensely over the years. There have been a lot of techniques described to fix these fractures. During the initial years of the 20th century, these fractures were primarily treated conservatively. It was not until 1970 that the emerging Arbeitsgemeinschaft fur Osteosynthesefragen (AO) principles were applied to fix these fractures [15-18]. But despite the various treatment options that have been available to date, these fractures still pose a challenge and are difficult to treat. The two most common fixation methods for fracture distal femurs are retrograde intramedullary nails and locking plates, but neither of them could prove its superiority over the other [4-7]. In a meta-analysis conducted by Jankowsky et al., they concluded that union rates among locking plates and retrograde intramedullary nails are similar [19]. The newer technique of combining nail and plate together was initially described for non-unions of the distal femur, where surgeons required a rigid construct for non-union management. Clinically, augmentation of long bone non-union previously treated with an intramedullary nail is not a totally novel approach. Its efficacy was proven by Birjandinejad et al. in 2009 in a series of 38 femoral and tibial non-unions primarily treated with IM nails. In their series, they concluded that 36 fractures, including all the femoral fractures, healed with the addition of a 4.5 mm compression plate construct with a nail in place [11]. Attum et al. conducted a retrospective study for non-union of the distal femur where a nail plate combination was used, and they also concluded that each patient in their study later progressed to union [9]. In a meta-analysis conducted by Quinzi et al., they did not find any significant differences in the frequency of major complications or re-operations in fractures treated with either locking plates, retrograde intramedullary nails, or distal femoral replacement, but they had different complication profiles [20]. Liporace and Yoon described favourable short-term outcomes in a total of 15 patients, with 6 being native and 9 being periprosthetic, with weight bearing initiated in the post-operative period, and found that all of their patients healed without infection, non-union, or hardware failures [10]. The idea behind the technique is that the nail-plate combination increases points of fixation and creates a fixed-angle construct while shifting the weight-bearing axis more medially along the anatomic axis, thereby conferring increased biomechanical stability with the benefits of both nailing and plating. In fractures in the elderly, osteopenic and/or obese that need early mobilisation, this nail plate combination is essential as it helps in the early rehabilitation of the patients, thereby minimising injury-related complications like DVT, pulmonary embolism, and pressure ulcers. They are also essential in cases of complex fractures with significant communication or segmental defects [9] and in inter- and peri-prosthetic fractures [21-23].

In our study, we used a nail plate combination in addition to implant removal with bone grafting in 10 patients with a non-union distal femur and broken implants. All the patients progressed to union with a minimum follow-up of 12 months and good functional results. Among the four patients who presented with a broken plate, our operative technique involved the use of the same skin incision and soft tissue access as the previous surgery, thereby limiting the soft tissue alteration. Due to the use of an adjunct intramedullary implant, post-operative rehabilitation could be pursued more rigorously and effectively. None of the follow-up radiographs showed any loss of reduction. The use of intramedullary implants as an adjunct also enabled early partial weight bearing in the patients. The outcomes of our study are comparable to the studies conducted by Attum et al. and Birjandinejad et al. [9,11]. As a relatively novel technique and in the absence of a comparative dual plating cohort in the study, it is unclear whether the results of the nail plate construct are superior or comparable to dual plating in the non-union of distal femoral fractures. Due to a relatively short duration of follow-up (12-24 months), our study was primarily based on clinico-radiological outcomes defined in terms of fracture union and IKDC score. A relatively short follow-up period and the limited number of patients participating in the study (10) are the primary drawbacks of our study.

## Conclusions

In our experience, this relatively novel technique of a combined nail plate construct offers encouraging outcomes in the management of non-union of distal femur fractures, especially in elderly and osteopenic patients. However, the study of long-term complications and comparative studies with existing modalities of treatment are required for a better application and understanding of this technique.
